# Radiation dose of computed tomography in pediatric head trauma imaging

**DOI:** 10.1007/s00234-025-03893-7

**Published:** 2026-01-19

**Authors:** Daniel Rosok, Marcel Opitz, Denise Bos, Yannick Thal, Marcel Drews, Raya Ocker-Serger, Mathias Holtkamp, Luca Salhöfer, Marcel Dudda, Johannes Haubold, Bernd Schweiger, Michael Forsting, Cornelius Deuschl, Sebastian Zensen

**Affiliations:** 1 Institute of Diagnostic and Interventional Radiology and Neuroradiology, University Hospital Essen, Essen, Germany; 2 Department of Trauma, Hand and Reconstructive Surgery, University Hospital Essen, Essen, Germany; 3 Department of Orthopedics and Trauma Surgery, BG Klinikum Duisburg, Duisburg, Germany

**Keywords:** Computed tomography, Emergency imaging, Pediatric trauma, Radiation safety

## Abstract

**Purpose:**

In emergency diagnostics, head CT and CT angiography (CTA) of craniocervical vasculature are indispensable for children, despite their increased radiation sensitivity. This study assesses the radiation dose metrics of head CT and CTA in pediatric patients managed in the trauma resuscitation unit (TRU).

**Methods:**

All patients aged 0–<15 years who underwent head CT and CTA in the TRU between April 2020 and August 2023 were included. CT dose index volume (CTDIvol) and dose-length product (DLP) were extracted from the Radimetrics Enterprise Platform, which also provided organ doses estimated via Monte Carlo simulations and effective doses (ED) derived from these estimates. Dose metrics were compared with national diagnostic reference levels (DRLs), defined for three pediatric age groups: I (0–<5 years), II (5–<10 years), and III (10–<15 years).

**Results:**

Of 212 pediatric TRU patients, 62.7% (133/212) underwent CT and 72.2% (96/133) received combined CT and CTA. Median CTDIvol and DLP increased with age, whereas ED decreased. For head CT, CTDIvol ranged from 18.9 mGy to 29.4 mGy, DLP from 282 to 460 mGycm, and ED from 1.6 to 1.3 mSv. For CTA, CTDIvol ranged from 1.4 to 2.2 mGy, DLP from 40 to 83 mGycm, and ED from 1.0 to 0.8 mSv. All doses remained below national DRLs.

**Conclusion:**

Head CT and CTA in pediatric trauma can be performed with radiation doses well below national DRLs. Careful dose management is important to reduce potential long-term cancer risks and deterministic effects such as lens cataract formation.

**Supplementary Information:**

The online version contains supplementary material available at 10.1007/s00234-025-03893-7.

## Introduction

Trauma is a leading cause of childhood mortality, accounting for about 20% of pediatric deaths [1, 2]. Traumatic brain injury is a primary reason for treatment in the trauma resuscitation unit (TRU), making computed tomography (CT) the preferred modality to diagnose injuries. Children, however, are more vulnerable to radiation-induced long-term cancer risks, particularly brain tumors and leukemia [3–6]. Head CT remains the gold standard for diagnosing intracranial hemorrhages and skull fractures, while CT angiography (CTA) detects potential vessel injuries and excludes cervical spine fractures. However, most head injuries in children are mild and do not require neurosurgical intervention, highlighting the need to balance diagnostic accuracy with minimizing radiation exposure. While MRI and ultrasound offer radiation-free alternatives, factors like scan duration, need for sedation, and reduced diagnostic sensitivity can limit their use in the acute setting [7]. Recent data on organ-equivalent and estimated effective doses (ED) in pediatric trauma patients undergoing combined head CT and CTA remain scarce; most prior studies have been limited to reporting organ or effective doses only, and rarely focus on children presenting through the TRU [8–10]. The objective of this study is to provide comprehensive data on radiation exposure from head CT and CTA in pediatric trauma patients across different age groups. This should be pursued through detailed, simulation-based estimation of both effective and organ-equivalent dose, supporting institutional and system-level quality improvement in pediatric head trauma imaging.

## Materials and methods

This retrospective, single-center observational study was approved by the local ethics committee. We included all pediatric patients ≤ 15 years with traumatic head injury who were managed through our TRU between April 2020 and August 2023 and underwent combined head CT and CTA on the same CT scanner. Patients > 15 years, those not managed via the TRU, and cases with incomplete head CT and CTA imaging were excluded. This primarily refers to patients who received head CT only, or a non-contrast head CT together with a non-contrast cervical spine examination. At our institution, children are routed through the TRU based on decisions by the emergency physician in consultation with the trauma surgical team, typically including patients with high-impact trauma or impaired consciousness. Clinical records were reviewed to determine reasons for TRU admission. Given that head CT is often indispensable in severe pediatric trauma for rapid exclusion of intracranial bleeding, the clinical necessity of concomitant CTA is less well defined. To contextualize CTA use in this cohort and to relate radiation exposure to diagnostic yield, we therefore assessed how many CTA examinations revealed vascular injury or other positive findings.

Diagnostic reference levels (DRLs) from the Federal Office for Radiation Protection in Germany exist for patients < 15 years of age [11]. Accordingly, patients were divided into three age groups: I (0 – <5 years), II (5 – <10 years), and III (10 – <15 years).

All examinations were performed on a 128-slice, single-source CT scanner (SOMATOM Definition Edge, Siemens Healthineers, Erlangen, Germany). Our standard protocol for head and craniocervical vasculature imaging consists of a non-contrast head CT followed by an arterial-phase CTA from the aortic arch to the top of the skull. In our university hospital neuroradiology department, any limitation in subjective image quality is routinely documented in the first line of the radiology report, and examinations are evaluated by board-certified neuroradiologists with several years of experience in pediatric trauma imaging. All included examinations underwent assessment of diagnostic image quality by reviewing the radiology report for such remarks. Table 1 details the imaging parameters. For the CTA protocols, quality reference mAs (QRM) values were relatively comparable across pediatric age groups and the adult protocol. However, the resulting CTDIvol values differed notably. This discrepancy arises primarily from the use of age-dependent tube voltage settings, which affect the output dose, and, in the adult protocol, differences in bowtie filter configuration.

**Table 1:** Technical parameters of pediatric head CT and CT angiography scans

Acquisition parameters	
Tube voltage (kV)	Automated tube voltage selection (CARE kV, Siemens Healthineers, Erlangen, Germany) was activated, permitting voltage settings between 70 and 120 kV. In practice, head CT was performed at 100 kV across all pediatric age groups, whereas CTA used 70 kV in group I (0–<5 years), 80 kV in groups II (5–<10 years) and III (10–<15 years) and 120 kV in the adult protocol
Tube current (mAs)	Activated automated tube current modulation (CARE Dose 4D, Siemens Healthineers, Erlangen, Germany)
Quality Reference mAs (QRM)	Head CT in age group I: 350 mAs (reference CTDIvol: 30 mGy), II: 384 mAs (reference CTDIvol: 33 mGy), III: 430 mAs (reference CTDIvol: 36 mGy), adult protocol: 538 mAs (reference CTDIvol: 46 mGy); CTA in age group: I: 205 mAs (reference CTDIvol: 1.9 mGy), II and III: 156 mAs (reference CTDIvol: 2.3 mGy), adult protocol: 200 mAs (reference CTDIvol: 13.5 mGy).
Rotation time (sec/ rotation)	1 (head CT), 0.5 (CTA)
Collimation (mm)	128 × 0.6 mm (virtual collimation from the flying focal spot); true collimation: 64 × 0.6 mm = 38.4 mm
Pitch	0.6 (head CT), 1.4 (CTA)
Reconstruction parameters	
Reconstruction type	Iterative reconstruction on level 3 (ADMIRE, Siemens Healthineers, Erlangen, Germany)
Matrix size/ pixel no.	512 × 512
Reference phantom for CTDIvol	
Head CT	16 cm head phantom
CTA	32 cm body phantom

Radiation exposure parameters, including CT dose index volume (CTDIvol), dose-length product (DLP), estimated ED, and organ-equivalent doses, were extracted from the Radimetrics Enterprise Platform (Version 3.4.2, Bayer, Leverkusen, Germany). For head CT, CTDIvol values referenced a 16 cm head phantom, while CTA values referenced a 32 cm body phantom. Organ-equivalent doses were estimated using automated Monte Carlo simulations, and technical acquisition parameters (tube voltage and tube current) were directly transferred from the connected CT system. The software selects the most appropriate reference phantom from a library of 18 Cristy-based anthropomorphic models, based on patient characteristics such as age, sex, and effective diameter [12]. The effective diameter was derived from the lateral topogram obtained prior to the scan. Phantom dimensions are then adjusted to match the patient’s effective diameter through interpolation, allowing individualized organ-equivalent dose estimation. The estimated ED was calculated by Radimetrics applying International Commission on Radiological Protection (ICRP) Publication 103 tissue-weighting factors to the simulated organ-equivalent doses [13, 14]. According to ICRP Publication 103, specific tissue-weighting factors are assigned to individual organs [14]. Organs without an individual weighting factor were included in the “remainder tissues” category, which is assigned a collective weighting factor [14]. Dose values therefore represent phantom-based estimates rather than true patient-specific measurements. Monte Carlo–based organ dosimetry has been validated in several studies and correlates well with physical dose measurements, but inherent uncertainties remain due to phantom selection, patient size approximation, and model-based assumptions [13, 15]. Organ-equivalent doses were reported only when values exceeded 1 mSv and for organs located within the primary beam or considered radiation-sensitive.

Data were analyzed using Statistical Package for Social Sciences Version 27.0 (SPSS, IBM Corp, Armonk, New York, USA; RRID: SCR_016479). The Shapiro-Wilk test was used to assess data distribution. As the variables did not follow a normal distribution, non-parametric data are reported as median and interquartile range (IQR). Dose parameters across age groups were compared using the Kruskal-Wallis test, followed by Dunn-Bonferroni post-hoc analysis, with *p* < 0.05 considered statistically significant. Figures were created using GraphPad Prism Version 5.0 (GraphPad Software, La Jolla, California, USA; RRID: SCR_002798).

## Results

### Clinical characteristics and CT imaging

During the study period, a total of 212 pediatric patients aged 0 – <15 years were treated in our TRU. All patients underwent a clinical examination and a Focused Assessment with Sonography for Trauma (FAST) in the initial diagnostic work-up. Subsequently, 62.7% (133/212) received a CT scan. Among these, 72.2% (96/133) underwent combined head CT and CTA. In the 37.3% (79/212) of cases without CT imaging, the primary reason was an interdisciplinary decision to pursue clinical observation or a stepwise diagnostic approach.

In 75.7% (28/37) of the excluded examinations, no standard protocol was performed; these were predominantly head CT without CTA. Studies that included additional phases (e.g. venography) were also excluded to avoid protocol-driven heterogeneity, which would otherwise bias dose estimates upward and limit comparability with standard trauma protocols.

**Fig. 1:**
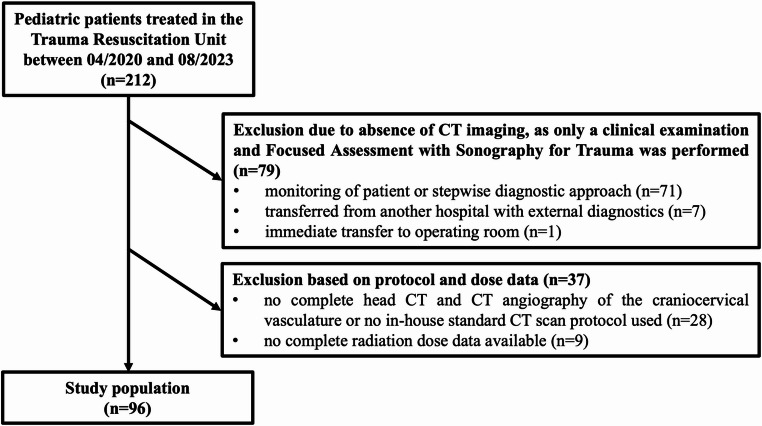
Flowchart of the assignment of the study population

In a smaller subset (24.3%, 9/37), radiation dose data was incomplete. No examination required exclusion on the basis of radiology report comments indicating insufficient diagnostic image quality. The median age of patients undergoing CT imaging was 8.0 years (IQR: 4.7–11.1 years), and 33.3% (32/96) of these patients were female. Figure 1 illustrates the assignment of the study population.

Head CT and CTA were predominantly performed due to traffic-related (47.9%, 46/96) and non-car-related trauma (49.0%, 47/96). Most frequent indications were traffic collisions with the child as a pedestrian (29.2%, 28/96) and falls from height or stairs (28.1%, 27/96). Non-traumatic indications were rare, accounting for only 3.1% (3/96) of cases (Table 2).

The analysis of clinically relevant CTA findings, revealed two pediatric patients with vascular injury (2.1%; 2/96), both representing traumatic dissections of the anterior carotid artery. One patient was 2 years old and presented with a fall from a third-floor balcony; the other was 5 years old and experienced high-velocity trauma with direct head impact against a pole. In addition, two further patients (2.1%; 2/96) demonstrated injuries of the cervical spine without evidence of vascular injury. In one case, bony reconstructions from the CTA showed cervical vertebral body fractures in a 12-year-old patient following a mountain-bike accident with an estimated jump distance of approximately 4 m. In the other case, bony reconstructions from the CTA demonstrated increased atlas–axis distance in a 3-year-old passenger involved in a motor vehicle collision; the suspected ligamentous distraction injury was subsequently confirmed on MRI. As outlined in the Materials and Methods section, this study only included patients who underwent combined head CT and CTA of the craniocervical vasculature; patients who had only non-contrast head CT and non-contrast cervical spine imaging were excluded.

**Table 2:** Causes of trauma leading to pediatric head CT and CT angiography scans

Causes of trauma leading to pediatric CT scans	% (n) of patients (total *n* = 96)
Traffic accidents	**47.9% (46/96)**
Pedestrian	29.2% (28/96)
Vehicle passenger	9.4% (9/96)
Cyclist	7.3% (7/96)
Scooter (motorized or non-motorized)	2.1% (2/96)
Non-car-related high- and low-energy trauma	**49.0% (47/96)**
Fall from height or stairs	28.1% (27/96)
Blunt impact trauma	9.4% (9/96)
Bicycle or scooter fall	7.3% (7/96)
Horse-related trauma	4.2% (4/96)
Internal medical or neurological emergencies	**3.1% (3/96)**
Post-resuscitation	1.0% (1/96)
Seizure	1.0% (1/96)
Transfer from another hospital	1.0% (1/96)

### Radiation dose analysis

**Fig. 2:**
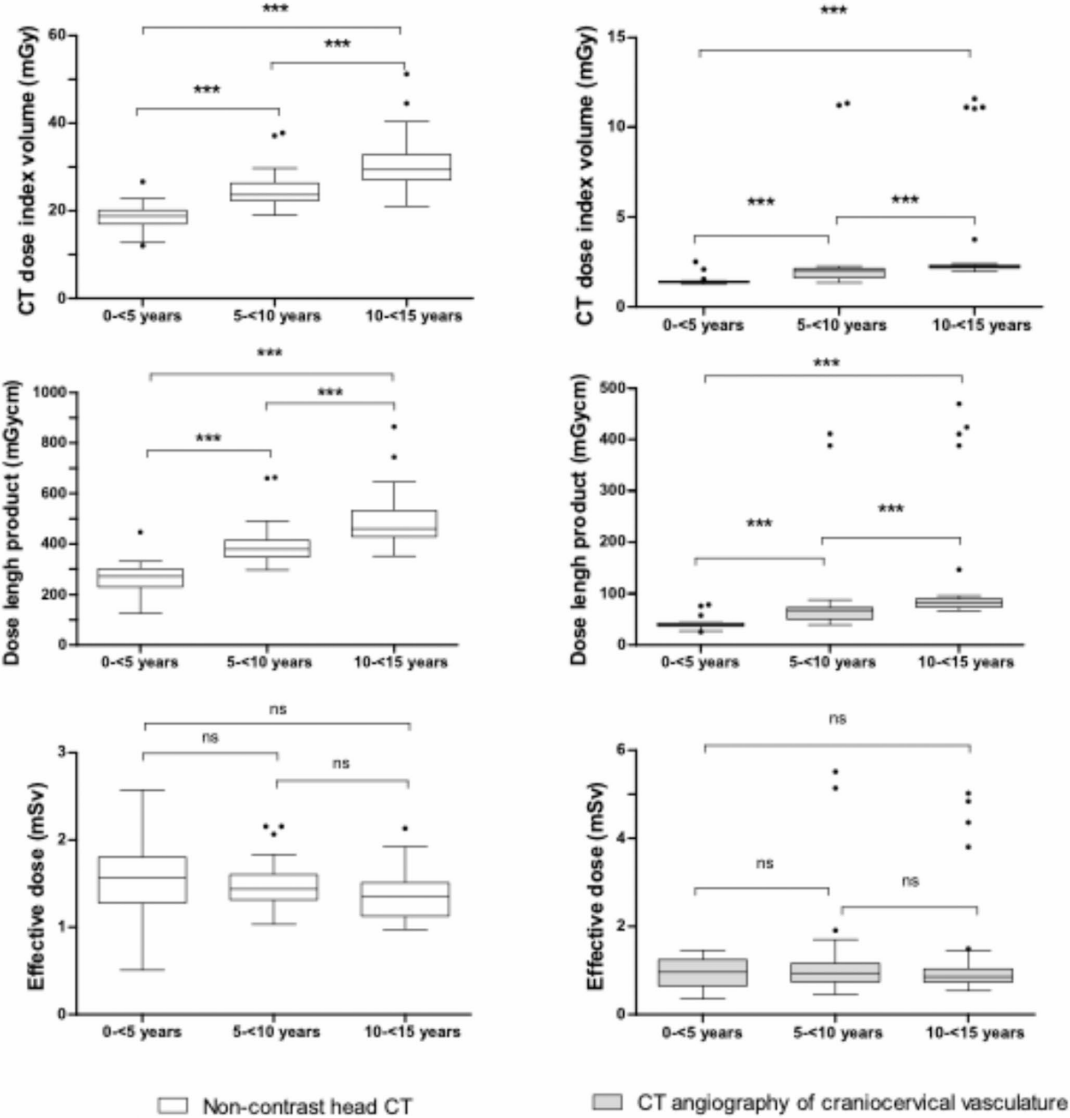
Radiation dose parameters of head CT and CT angiography of craniocervical vasculature in pediatric trauma patients by age group. Whiskers follow Tukey’s method. *** indicates *p* < 0.001; ns = not significant. Data points plotted as individual dots represent outliers, which were predominantly examinations performed using adult protocols

Radiation dose analysis was performed for all patients who underwent standard-protocol head CT and CTA scans with complete dosimetric data (*n* = 96). CTDIvol was referenced to the 16 cm CTDI phantom for head CT and to the 32 cm CTDI phantom for CTA. We observed that CTDIvol and DLP increased significantly (*p* < 0.001) with advancing pediatric age group, whereas ED decreased marginally (*p* > 0.05) for both head CT and CTA (Fig. 2; Table 3).

**Table 3:** Radiation dose metrics for head CT and CT angiography of craniocervical vasculature in pediatric trauma patients

CT scan area	Radiation dose parameter		Age group		
			I (0 – <5 years) *n* = 26	II (5 – <10 years) *n* = 37	III (10 – <15 years) *n* = 33
Head CT	German DRL^a^ for CTDIvol^b^ (mGy)		35	40	45
	CTDIvol (mGy)	Median	18.9	23.9	29.4
		IQR^c^	17.5–20.1	22.3–26.2	27.1–32.5
	DLP^d^ (mGycm)	Median	282	381	460
		IQR	253–301	351–413	431–533
	ED^e^ (mSv)	Median	1.6	1.4	1.3
		IQR	1.3–1.8	1.3–1.6	1.1–1.5
CT angiography of the craniocervical vasculature	CTDIvol (mGy)	Median	1.4	2.0	2.2
		IQR	1.4–1.5	1.5–2.1	2.2–2.3
	DLP (mGycm)	Median	40	68	83
		IQR	37–43	50–72	75–88
	ED (mSv)	Median	1.0	0.9	0.8
		IQR	0.7–1.2	0.8–1.2	0.7–1.0

^a^Diagnostic reference level (DRL) for head CT, ^b^CT dose index volume (CTDIvol) referenced to the 16 cm CTDI phantom for head CT and to the 32 cm CTDI phantom for CT angiography, ^c^interquartile range (IQR), ^d^dose-length product (DLP), ^e^estimated effective dose (ED)

**Fig. 3:**
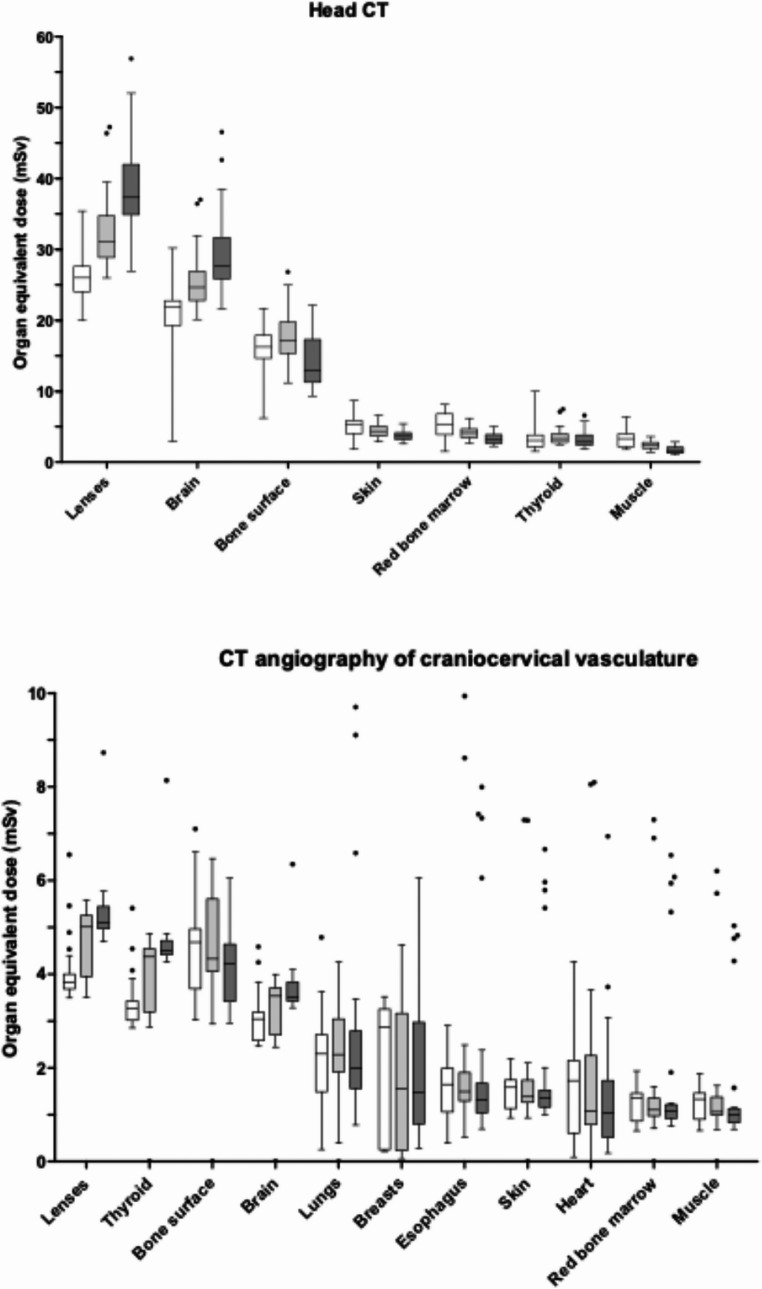
Organ-equivalent doses of head CT and CT angiography of craniocervical vasculature in pediatric trauma patients. Whiskers are defined according to Tukey’s method. Data points plotted as individual dots represent outliers, which were predominantly examinations performed using adult protocols. Box-plot colors correspond to the three age groups: white for group I (0–<5 years), light grey for group II (5–<10 years), and dark grey for group III (10–<15 years)

17.7% (17/96) of patients were not scanned using age-appropriate protocols. In the subanalysis of radiation exposure (Table 4), protocols intended for older age groups or adults consistently yielded higher doses than age-appropriate protocols, whereas protocols derived from younger age groups yielded lower doses. This effect was most pronounced when adult protocols were inadvertently applied in children, resulting in substantially increased doses—for example, the median CTDIvol for head CT in age group II was 23.9 mGy with an age-appropriate protocol versus 37.5 mGy with an adult protocol. Nevertheless, all median CTDIvol values for head CT remained well below the national DRLs. The outliers in Figs. 2 and 3 that represent comparatively higher doses correspond to examinations performed with non-age-appropriate or adult protocols.

**Table 4:** Radiation exposure parameters in patients with non–age-appropriate CT protocols

CT scan area	Radiation dose parameter		Age group						
			I (0 – < 5 years) scanned with protocol of II *n* = 1	II (5 –<10 years) scanned with protocol of I *n* = 4		II (5 – <10 years) scanned with protocol of III *n* = 3	II (5 – <10 years) scanned with protocol of adult *n* = 2	III (10 –<15 years) scanned with protocol of II *n* = 3	III (10 – <15 years) scanned with protocol of adult *n* = 4
Head CT	CTDIvol^a^ (mGy)	Median	26.6	23.1		27.1	37.5	25.3	39.2
		Range	-	19.7 − 29.7		22.8 − 28.6	37.2 − 37.8	21.0–32.1	33.6 − 51.2
	DLP^b^ (mGycm)	Median	446	355		389	662	411	633
		Range	-	311–416		345 − 482	660 − 664	352–533	450–865
	ED^c^ (mSv)	Median	1.8	1.5		1.5	2.2	1.3	1.6
		Range	-	1.4 – 1.8		1.3 – 1.6	2.1–2.2	1.1 – 1.7	1.3 – 2.1
CT angiography of the craniocervical vasculature	CTDIvol (mGy)	Median	2.1	1.5		2.1	11.3	2.0	11.1
		Range	-	1.4 – 2.2		2.1 – 2.2	11.2 − 11.3	2.0 – 2.1	11.0 − 11.6
	DLP (mGycm)	Median	76	47		75	400	73	417
		Range	-	43 – 64		71 – 78	388 − 411	72 – 73	388–469
	ED (mSv)	Median	1.5	0.8		1.0	5.3	1.0	4.6
		Range	-	0.6 – 1.2		0.9 – 1.1	5.1–5.5	0.9 – 1.0	3.8 – 5.0

^a^CT dose index volume (CTDIvol) referenced to the 16 cm CTDI phantom for head CT and to the 32 cm CTDI phantom for CT angiography, ^b^dose-length product (DLP), ^c^effective dose (ED)

### Organ equivalent doses of pediatric head CT and CT angiography

Analysis of organ-equivalent doses for head CT and CTA showed that the lenses, brain, bone surface, and thyroid received the highest median doses (Fig. 3; Supplement Tables 1 and 2). Lens doses were highest overall and increased across age groups in both modalities. For CTA, the radiation-sensitive thyroid also demonstrated comparatively elevated doses. In contrast, doses to red bone marrow, muscles, and skin were lower and generally decreased with advancing age group, with muscles receiving the lowest values.

## Discussion

This study provides new insights by offering trauma-specific benchmarking of pediatric head CT and CTA, integrating both examination types, and by presenting a detailed analysis of estimated effective and organ-equivalent doses derived from the radiation dose monitoring platform Radimetrics. Our analysis yielded five key findings: First, the most common indications for head CT und CTA following trauma were motor vehicle accidents and falls. Second, the prevalence of positive vascular injury findings on CTA was 2.1%, consisting of traumatic carotid artery dissections. Third, head CT and CTA in pediatric trauma can be performed with radiation doses well below national DRLs. Forth, age-based stratification of ED revealed an inverse relationship between patient age and radiation exposure, with younger children being associated with higher estimated ED. Fifth, organ-equivalent dose analysis showed that the lenses were the most highly exposed organ in head CT and CTA.

Head trauma is among the leading causes of morbidity and mortality in children in developed countries [16]. This is attributable to the distinct anatomical and biomechanical characteristics of pediatric patients, including a disproportionately large skull relative to overall body mass and underdeveloped neck musculature. Additionally, in infants, open fontanelles allow greater force transmission to the brain parenchyma, and cerebral vessels remain immature with impaired autoregulation after severe trauma [17, 18]. Head CT remains central in the evaluation of pediatric patients with suspected traumatic brain injury following treatment in the TRU. Substantial variability exists in the utilization of CT for pediatric head trauma, ranging from approximately 20–70% in the U.S [16]. The relatively high overall CT rate of 62.7% and combined head CT and CTA rate of 45.3% in our cohort likely reflect the study population, as only children with severe trauma are managed through our TRU, whereas mild head injuries are typically treated in the pediatric emergency department and thus not included. At our supra-regional level I trauma center, CTA in addition to head CT has traditionally been part of the TRU protocol to exclude vascular injuries in high-impact trauma or cases with unclear neurological findings. Institutional data showing a high proportion of severely injured pediatric patients (32% with Injury Severity Score ≥ 16, mortality of 20.3%) further supports our cautious diagnostic approach [2]. In cases of severe traumatic brain injury, CTA is essential for excluding vascular injuries and producing multiplanar reconstructions focused on bony structures. Arterial dissection was identified in 2.1% of CTA examinations, representing a low but clinically meaningful diagnostic yield. This underscores the importance of carefully balancing the potential diagnostic benefit of CTA against the associated radiation exposure, advocating for its selective application in high-risk scenarios and strict adherence to pediatric dose standards [19]. The most frequent indications for head CT and CTA in our TRU were severe injury resulting from motor vehicle collisions (47.9%) and falls from significant heights or down staircases (49.0%). The high proportion of trauma-related head CT and CTA in our cohort reflects the trauma-focused nature of the TRU. Non-traumatic neurological and internal medicine cases are typically routed through other departments at our institution, accounting for their low representation and the correspondingly lower imaging frequency.

In a British study, the median CTDIvol across comparable pediatric age groups ranged from 21.2 to 37.5 mGy, and in an Italian study from 27 to 51 mGy, whereas in our cohort, values ranged from 18.9 to 29.4 mGy [20, 21]. Similarly, our median CTDIvol and DLP were lower than those reported in the largest U.S. study on pediatric DRLs [22]. In their < 6 years group, the median CTDIvol was 25 mGy compared with 18.9 mGy in our < 5 years cohort, and the median DLP was 409 mGycm versus 282 mGycm in our study [22]. Our estimated effective and organ-equivalent doses were comparable to those reported by Kiani et al., who also used Monte Carlo simulations [10]. Stratification of ED by age revealed an inverse relationship between patient age and estimated ED, consistent with other studies demonstrating younger children being associated with higher estimated ED [23–25]. Although partial differences in age stratification across studies limit direct comparisons, our findings suggest that radiation doses for head CT in our study are generally within or below previously reported reference ranges. All mentioned CTDIvol values were referenced to the 16 cm diameter CTDI phantom. Organ dose analysis revealed that, during head CT, the lenses and brain received the highest exposures, with equivalent doses of up to 37.3 mSv. For CTA, the lenses, bone surface, and thyroid were most affected, with doses reaching 5.1 mSv. The thyroid received comparable organ-equivalent doses from both head CT and CTA, highlighting its consistent radiosensitivity across protocols. The lens is primarily susceptible to deterministic effects, such as radiation-induced cataract formation, whereas the thyroid is at risk for stochastic effects, including radiation-induced malignancy. Nonetheless, radiological examinations should not be withheld when clinically indicated, as doing so may lead to avoidable complications. The variability in the ED/DLP ratio across age groups and between head and CTA scans is expected and reflects differences in organ doses and tissue weighting derived from the Monte Carlo simulations. Notably, although DLP values were higher for head CT than for CTA, the ED values were comparable to CTA due to its higher ED/DLP ratio.

All dose data for head CT remained well below German DRLs. Currently, Germany lacks DRL for CTA in the pediatric population. Our cohort of 96 pediatric patients, with age-stratified data across three age groups, may offer valuable insights and help guide future investigations. Presently, German guidelines for pediatric polytrauma management do not include the use of CTA in children trauma [17]. Given the high trauma severity in our cohort, we deliberately choose performing CTA of craniocervical vasculature in individual cases to avoid diagnostic oversights. In our view, the appropriate scan extent in pediatric patients remains a subject of discussion and should ideally be defined by interdisciplinary consensus within the trauma team. This underscores the essential role of pediatric-trained radiologists in the TRU. Notably, 17.7% of patients in our study cohort were not examined using age-appropriate protocols, indicating a gap in protocol adherence. Potential reasons include the time-critical nature of emergency care and the challenges of accurately estimating a child’s age or body habitus—factors that may lead to protocol selection errors in acute settings. Following this study, the trauma team has considered the practical implications of these findings. All pediatric CTAs and their associated radiation doses are now systematically reviewed in interdisciplinary trauma meetings to support more stringent and explicit decision-making regarding CTA indications in children at our TRU.

Studies investigating the potential cancer risk associated with diagnostic imaging in children have reported mixed results. While some studies suggest an increased risk of malignancies, such as brain tumors and leukemia, following exposure to CT radiation in childhood, other studies have not found a clear or consistent association [4, 26–29]. These divergent findings highlight the ongoing uncertainty in quantifying long-term cancer risk from pediatric imaging and underscore the importance of careful radiation management. Organ-based dose modulation, which reduces anterior beam intensity during head CT, can lower exposure to radiosensitive organs such as the lenses [30]. Additional protective measures, including thyroid and lens shielding, may also reduce radiation doses but can be impractical or even counterproductive in acute pediatric emergencies due to limited patient cooperation. Modern iterative reconstruction algorithms—such as the ADMIRE technique used in this study—constitute a key strength of the applied CT protocols. Iterative reconstruction improves signal-to-noise characteristics and reduces image noise at lower tube currents, thereby maintaining diagnostic image quality at lower radiation doses [31, 32]. Additional strategies for dose reduction in pediatric trauma imaging include weight- or size-adapted protocols, automated exposure control, and emerging AI-based denoising methods. Weight- or size-based parameterization enables individualized adjustment of tube current and voltage to patient habitus, while automated exposure control dynamically modulates tube current according to tissue attenuation both along the z-axis and in the gantry rotation direction [33–35]. AI-assisted reconstruction and post-processing may further decrease dose requirements by suppressing noise in low-dose acquisitions while preserving diagnostic detail [36]. These approaches reflect the principles of justification and optimization within the “as low as reasonably achievable” (ALARA) framework and align with ongoing initiatives by the American College of Radiology (ACR) and the American Association of Physicists in Medicine (AAPM) to promote evidence-based and judicious use of CT in pediatric emergency care [37–41].

Our study is not without limitations. It was conducted at a single TRU with specific scan protocols, which may limit the generalizability of our findings, but also allows for a detailed analysis of these protocols and its effects on radiation doses. Deviations from the intended scan range—whether over- or under-scanning—can affect the accuracy of the dose estimates obtained in this study. Organ doses were provided by the software only in mSv, and extraction in mGy was not possible in Radimetrics, which may limit comparability with organ dose data reported in previous studies. The values therefore represent organ-equivalent doses, and this terminology is used consistently throughout the manuscript.

## Conclusion

Head CT in pediatric trauma can be performed with radiation exposure levels consistently below national DRLs. The observed inverse correlation between age and estimated ED underscores the importance of pediatric tailored imaging strategies with adjusted imaging parameters and specialized protocols. Given the lack of DRLs for pediatric CTA in Germany, our data could provide insights that could encourage similar analyses to enhance radiation safety in this high-risk population. However, further data from national institutions are needed to strengthen the validity.

## Supplementary Information

Below is the link to the electronic supplementary material.

Supplementary Material 1

## Data Availability

The data that support the findings of this study are not openly available due to reasons of sensitivity and are available from the corresponding author upon reasonable request.
