# Supplementary material for: Radiation dose of computed tomography in pediatric head trauma imaging
Source: Neuroradiology. 2026 Jan 19;68(2):339–49. doi: 10.1007/s00234-025-03893-7 (PMC13021731; doi:10.1007/s00234-025-03893-7)
Supplement: Supplementary file 1 — Supplementary Material 1 [file 234_2025_3893_MOESM1_ESM.docx]

**Supplement Table 1** Organ equivalent doses for head CT in pediatric trauma patients

| Organ equivalent doses (mSv) for non-contrast head CT scan | | | | | | |  |
| --- | --- | --- | --- | --- | --- | --- | --- |
|  | **Age group I (0** – **<5 years)** | | **Age group II (5** – **<10 years)** | | **Age group III (10** – **<15 years)** | |  |
| Organs | **Median** | **Interquartile range** | **Median** | **Interquartile range** | **Median** | **Interquartile range** |  |
| Lenses | 26.0 | 24.2 – 27.3 | 31.0 | 29.1 – 34.5 | 37.3 | 35.1 – 41.9 |  |
| Brain | 21.9 | 19.4 – 22.7 | 24.6 | 23.0 – 26.9 | 27.7 | 25.9 – 31.5 |  |
| Skin | 5.3 | 4.0 – 5.8 | 4.4 | 3.8 – 5.1 | 3.6 | 3.3 – 4.1 |  |
| Bone surface | 16.2 | 14.7 – 17.7 | 17.1 | 15.4 – 19.6 | 12.9 | 11.6 – 17.2 |  |
| Red bone marrow | | 5.3 | 3.9 – 6.7 | 4.1 | 3.5 – 4.6 | 3.1 | 2.6 – 3.9 |
| Thyroid | 3.0 | 2.2 – 3.7 | 3.3 | 2.8 – 3.9 | 2.8 | 2.4 – 3.7 |  |
| Muscles | 3.3 | 2.2 – 3.9 | 2.3 | 2.0 –2.7 | 1.5 | 1.3 – 2.1 |  |

**Supplement Table 2** Organ equivalent doses for CT angiography of the craniocervical vasculature in pediatric trauma patients

| Organ equivalent doses (mSv) for CT angiography of the craniocervical vasculature | | | | | | |
| --- | --- | --- | --- | --- | --- | --- |
|  | **Age group I (0** – **<5 years)** | | **Age group II (5** – **<10 years)** | | **Age group III (10** – **<15 years)** | |
| Organs | **Median** | **Interquartile range** | **Median** | **Interquartile range** | **Median** | **Interquartile range** |
| Lenses | 3.8 | 3.7 – 4.0 | 5.0 | 4.3 – 5.2 | 5.1 | 5.0 – 5.4 |
| Brain | 3.0 | 2.6 – 3.2 | 3.5 | 2.7 – 3.7 | 3.5 | 3.4 – 3.8 |
| Skin | 1.6 | 1.2 – 1.7 | 1.4 | 1.3 – 1.7 | 1.4 | 1.2 – 1.5 |
| Heart | 1.7 | 0.7 – 2.1 | 1.1 | 0.8 – 2.0 | 1.0 | 0.5 – 1.7 |
| Lungs | 2.3 | 1.6 – 2.7 | 2.3 | 1.9 – 3.0 | 2.0 | 1.6 – 2.8 |
| Breasts | 2.9 | 0.3 – 3.2 | 1.6 | 0.3 – 3.1 | 1.5 | 1.0 – 2.5 |
| Muscles | 1.3 | 0.9 – 1.4 | 1.1 | 1.0 – 1.4 | 1.0 | 0.8 – 1.1 |
| Red bone marrow | 1.4 | 0.9 – 1.5 | 1.1 | 1.0 – 1.3 | 1.1 | 0.9 – 1.2 |
| Thyroid | 3.3 | 3.0 – 3.4 | 4.4 | 3.3 – 4.5 | 4.5 | 4.4 – 4.7 |
| Bone surface | 4.7 | 3.8 – 5.0 | 4.3 | 4.1 – 5.5 | 4.2 | 3.4 – 4.6 |
| Esophagus | 1.6 | 1.1 – 1.9 | 1.5 | 1.3 – 1.9 | 1.3 | 1.0 – 1.6 |
